# Comparison of Vertebral Artery and Middle Cerebral Artery Monitoring for Right-to-left Shunt Detection by Contrast-enhanced Transcranial Doppler

**DOI:** 10.1038/srep24932

**Published:** 2016-04-21

**Authors:** Yu-Zhu Guo, Yong-Sheng Gao, Zhen-Ni Guo, Peng-Peng Niu, Yi Yang, Ying-qi Xing

**Affiliations:** 1 Neuroscience Center, Department of Neurology, The First Hospital of Jilin University, Changchun, China; 2 Department of Cardiac Surgery, The First Hospital of Jilin University, Changchun, China

## Abstract

Contrast-enhanced transcranial Doppler (c-TCD) is a reliable and reproducible method for right-to-left shunt (RLS) detection, with high sensitivity. Monitoring the middle cerebral artery (MCA) is an optimal choice, yet for patients with insufficient temporal bone windows or severe stenosis of carotid arteries, an alternative should be established. The aim of the present study was to further establish whether c-TCD with vertebral artery (VA) monitoring is as effective as MCA monitoring for RLS detection. We evaluated 194 subjects for RLS detection with VA and MCA monitoring simultaneously. There was no significant difference between the positive rates of VA and MCA monitoring for RLS detection. c-TCD with VA monitoring could be an alternative for RLS detection, with high sensitivity and specificity both at rest and during the Valsalva manoeuvre.

Right-to-left shunt (RLS) has been recently reported in a number of clinical conditions, such as migraine, platypnea-orthodeoxia, obstructive apnea, and decompression sickness^1^. The presence of RLS is a well-known risk factor for cryptogenic stroke^2^. Related ischemic lesions are located predominantly in the vertebrobasilar circulation (VBC), especially in patients with provoked RLS^3^. To date, contrast-enhanced transcranial Doppler (c-TCD) has been widely used in detecting both cardiac and extracardiac RLS, as it is more reliable and reproducible with higher sensitivity compared with invasive transesophageal echocardiography (TEE)^4^,^5^. However, RLS detection is limited by insufficient temporal bone windows in 10–20% of stroke patients^6^. Monitoring at the carotid artery might be considered a possible alternative method, but it has not been included in broad clinical practice^7^,^8^. c-TCD with vertebrobasilar monitoring has also been considered and compared for detecting RLS^9^,^10^. The aim of our study was to further establish whether c-TCD with vertebral artery (VA) monitoring is as effective as middle cerebral artery (MCA) monitoring for RLS detection in a Chinese population.

## Results

In total, 194 participants (38 ± 12.6 years; 125 women and 69 men) were included in the study. Demographic and clinical characteristics are detailed in Table 1.

### Positive rate of RLS detection

The total positive rates of RLS detection in the left middle cerebral artery (LMCA) and left vertebral artery (LVA) monitoring were 36.6% and 35.6%, respectively. For constant RLS, positive rates of RLS detection in LMCA and LVA monitoring were 21.6% and 20.6%, respectively. For a provoked RLS, positive rates of RLS detection in LMCA and LVA monitoring were both 14.9%. Although different results might be detected in the same patient, there was no significant difference between LMCA and LVA monitoring for RLS detection, including constant and provoked RLS. Besides, the sensitivity and specificity of LVA monitoring were relatively high, with the LMCA taken as the standard (Tables 2, 3, 4).

### Degree of RLS

Grade III RLSs were detected in 25 (12.9%) and 23 (11.9%) of the 194 patients who underwent the c-TCD procedure in LMCA and LVA monitoring, respectively. Grade II RLSs were detected in 13 (6.7%) and 11 (5.6%) patients in LMCA and LVA monitoring, respectively. The corresponding numbers for grade I RLSs were 33 (17.0%) and 34 (17.5%), respectively.

There was no significant difference between LMCA and LVA monitoring in terms of the degree of RLS detection, although the degree of RLS seemed higher in LMCA monitoring than in LVA monitoring (p = 0.079) (Table 5).

### Time of the first microbubble (MB) occurrence

Among the 194 patients, 36 exhibited MBs in both LMCA and LVA monitoring at rest, whereas 68 exhibited MBs in both LMCA and LVA monitoring during the Valsalva manoeuvre (VM). The mean time to first MB occurrence in LVA monitoring was slightly longer than that in LMCA monitoring both at rest (10.67 ± 4.18 s vs. 9.39 ± 3.71 s, p < 0.001) and during the VM (11.90 ± 3.89 s vs. 10.91 ± 3.93 s, p < 0.001), although this was not clinically significant.

## Discussion

The present study determined whether VA monitoring is as effective as MCA monitoring for RLS detection in a Chinese population. The main finding was that LVA monitoring could be used as an alternative for RLS detection, with high sensitivity and specificity both at rest and during VM.

The presence of RLS is a known risk factor for ischemic stroke, and related ischemic lesions are located predominantly in VBC, especially in patients with provoked RLS^3^. Besides, about 10–20% of stroke patients have insufficient temporal bone windows^6^. Thus, it is interesting whether RLS detection through the transoccipital window is as effective as MCA monitoring, or even better for provoked RLS, with c-TCD.

We once had a patient highly suspected to have RLS and whose c-TCD with LMCA monitoring was negative; however, VA monitoring exhibited curtain at rest and during VM, as his carotid arteries were severely narrow. Thus, for patients with severe carotid artery circulation stenosis, c-TCD with VA monitoring is necessary.

Del Sette *et al.* compared transtemporal and transoccipital approaches by c-TCD for RLS diagnosis. They chose the right middle cerebral artery (RMCA, with a depth of 45–55 mm) and VBC (75–85 mm) and found that RLS was present in the MCA of 28 subjects and VBC of 16 subjects at rest; the respective numbers were 43 and 36 after VM. No subject was RLS positive in VBC but negative in the MCA. The time to MB appearance was longer in the VBC recording, both at rest (5.5 ± 3.0 s vs. 8.25 ± 8.2 s, p = 0.01) and during VM (4.36 ± 1.7 s vs. 6.77 ± 2.5 s, p < 0.001)^10^. However, in our study, the LMCA (40–65 mm) and LVA (50–75 mm) were monitored simultaneously, and no significant difference was found between the RLS positive rates using each method. Moreover, the mean time to first MB occurrence in LVA monitoring was only slightly longer than that in LMCA monitoring. Additionally, we did have subjects who were RLS positive in VA monitoring but negative in MCA monitoring. All VA monitoring reached a high sensitivity for total, constant, and provoked RLS detection in our study, whereas Del Sette *et al.* showed a low sensitivity (57.14%) at rest. Their depth of arteries leading to the time difference of MBs arrival and their contrast agent (CA) (9 mL of saline solution and 1 mL of air without a drop of the patient’s blood) might be the two reasons for this difference, as blood might significantly increase the number of MBs and prolong the retention of MBs in the CA^11^.

Besides, the VBC at a depth of 75–85 mm could either be from the VA or the basilar artery (which may objectively enhance the diagnostic yield because MBs coming through both vertebral arteries can be detected in the basilar artery). One VA corresponding to one MCA would be more comparable^12^. Furthermore, VA monitoring is technically simpler than basilar artery monitoring. The left side of the MCA and VA was chosen instead of the right side, as it was more convenient for the operation. Besides, the left and right VAs are often asymmetrical, with left dominance occurring in 50% of cases, similarly sized VAs and right dominance each occurring in 25% of cases^13^.

Mitsumura *et al.* evaluated the MCA and VA separately for RLS using transcranial colour flow imaging in 52 subjects and showed that the evaluation of RLS in the intracranial VA has equal diagnostic ability to that in the MCA^14^. Recently, Perren *et al.* compared the monitoring of cervical arteries (submandibular internal carotid artery and VA) to that of the MCA, respectively^15^. They examined RLS in the supine position for the MCA and with the head turned in the lateral position for the VA (extra cranial V3 segment at a depth range of 25–35 mm) separately with a small sample of 43 subjects, while we simultaneously monitored the VA (intracranial V4 segment at a depth range of 50–75 mm) and MCA with a modified head frame in 194 subjects. The V4 segment is easier to monitor from a technical perspective while the V3 segment presents more anatomical variations^16^. The results of the present study and the two previous studies showed that VA monitoring is a valid screening method to detect RLS.

Paradoxical emboli have a particular propensity for posterior circulation, although the amount of blood in the vertebrobasilar system is lower (20% of the carotid circulation)^17^. Kim *et al.* suggested that the VM may lower the innervation of the sympathetic nervous system or provoke RLS and simultaneously increase blood flow to the VBC, thereby causing provoked RLS-related stroke to predominate in the posterior circulation^3^. However, LVA monitoring did not reveal more MBs than did LMCA monitoring in provoked RLS as we expected in our study, suggesting that a VM-induced increased blood flow with more emboli to the VBC might not be the crucial reason why stroke patients with provoked RLS were more likely to have ischemic lesions located in the posterior circulation.

There are several limitations in our study. Firstly, the VA might be lost or changed, especially during the VM; as the probe for VA monitoring was manually positioned, it could not be positioned the same as for MCA monitoring, in which the probe was fixed on the head frame. However, we reproduced the procedures to ensure that at least one procedure was valid for VA monitoring. The criterion of a “valid procedure” is that the spectrum of the M-mode remains stable. Secondly, the right lateral decubitus was not the optimal position for RLS detection, due to the anatomic location of the right atrium and patent foreman ovale, which may have affected the performance of the standard VM^18^,^19^,^20^,^21^. However, MCA and VA monitoring were performed simultaneously in the same position, even if the positive rate of RLS detection may be a little lower than that in other positions; however, the comparison between MCA and VA monitoring was not affected. Thirdly, we chose the left side of the MCA and VA instead of the right side, as it was more convenient for the operation, and RVA dominance accounts for only a quarter of cases^13^. The sensitivity of VA monitoring may be affected by the anatomical difference (the RVA branches out from the brachiocephalic artery, and the LVA directly branches out from the aortic arch) when using the right side of the VA for monitoring, which was not validated in our study. We suggest LVA monitoring for RLS detection in patients with insufficient temporal bone windows or significant artery stenosis in the carotid circulation. Fourthly, RLS confirmed by TEE was not mentioned because TEE was not accepted by the majority of patients.

c-TCD with LVA monitoring could be an alternative for RLS detection, albeit not better than MCA monitoring. RLS in patients with insufficient temporal bone windows or significant artery stenosis in the carotid circulation can be detected by c-TCD with VA monitoring.

## Methods

### Participants

From February to April 2015, we evaluated 318 consecutive subjects from the Department of Neurology at our hospital for RLS detection by c-TCD. Those who were unable to perform the standard VM because of severe heart or lung disease, or cognitive or coordination impairment (n = 13); subjects with an insufficient temporal window (n = 43); and follow-up patients who had undergone transcatheter patent foramen ovale closure (n = 24) were excluded from our study. After further excluding 44 subjects who presented with significant artery stenosis (n = 27) of the carotid or VBC or congenital vertebral artery dysplasia (n = 17), all of the remaining 194 subjects underwent c-TCD with VA and MCA monitoring simultaneously. The study design was approved by the ethics committee of The First Hospital of Jilin University, and all patients provided written informed consent. The methods were carried out in accordance with the approved guidelines.

### c-TCD protocol

**Figure 1:**
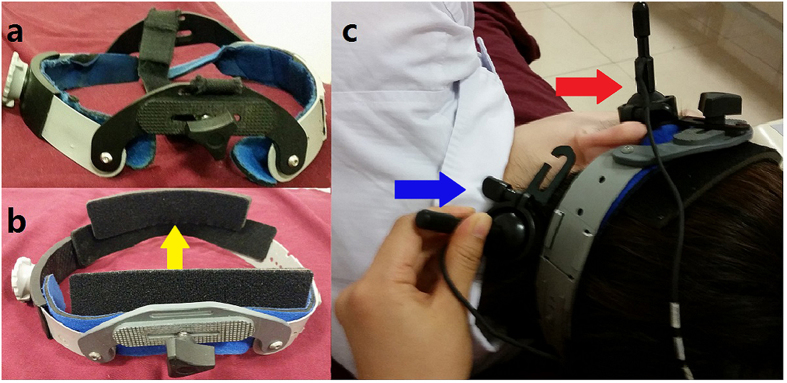
Modified head frame and simultaneous left middle cerebral artery (LMCA) and left vertebral artery (LVA) monitoring. (**a**) Normal head frame. (**b**) Modified head frame with a cushion stitched on the lateral inside, which allowed patients to lie on their right side comfortably. (**c**) Simultaneous LMCA and LVA monitoring. The yellow arrow points to the modification of the head frame. The blue arrow points to LVA monitoring with manual probe fixation. The red arrow points to LMCA monitoring with the modified head frame.

c-TCD examinations were performed using a Multi-DopX4 transcranial Doppler detector (DWL, Sipplingen, Germany), and both LMCA and LVA were monitored simultaneously. An 18-gauge needle was inserted into patients’ cubital vein. Mean flow velocity of the LMCA was recorded with a monitoring probe mounted in the modified head frame, which allowed patients to lie on their right side comfortably. The other probe was simultaneously and manually positioned in the corresponding transoccipital window for LVA monitoring (Fig. 1). A sample volume of 8 mm in length and a low gain provided the optimal setting for the background spectrum. The depth of monitoring was 40–65 mm and 50–75 mm for the MVA and VA, respectively. After training patients on the VM, the test was performed at rest and during the VM twice, the strength of which was measured by the peak of the Doppler MCA flow velocity curve. The CA was prepared using 9 mL of saline solution, 1 mL of air, and one drop of the patient’s blood, which was vigorously mixed between two 10-mL syringes via a three-way stopcock^11^. After 30 mixing cycles, the CA was injected rapidly as a bolus injection. VM started 5 s after the CA was injected, and it lasted for 10 s.

**Figure 2:**
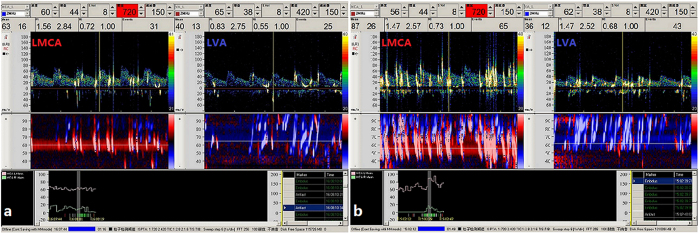
The spectra on contrast-enhanced transcranial Doppler (c-TCD) of simultaneous left middle cerebral artery (LMCA) and left vertebral artery (LVA) monitoring. (**a**) The spectra on c-TCD of simultaneous LMCA and LVA monitoring at rest. (**b**) The spectra on c-TCD of simultaneous LMCA and LVA monitoring during the Valsalva manoeuvre.

The monitored Doppler spectra were stored for offline analysis of the time and the number of MBs that occurred, which was used to assess the size and functional relevance of the RLS (Fig. 2). An MB was defined as a visible and audible (click, chirp, or whistle), short-duration, high-intensity signal within the Doppler flow spectrum. In our study, ultrasound was performed in M-mode to improve detection of the embolus track for the diagnosis of RLS. RLS was defined as constant or provoked RLS if MBs were detected at rest or only during the VM, respectively. There are several different methods of classification, but we adopted the four-level categorization of the International Consensus Criteria according to MB appearance in the TCD spectrum using unilateral MCA monitoring: negative, no occurrence of MBs; grade I, 1–10 MBs; grade II, >10 MBs, but no curtain; and grade III, curtain, that is, a single MB could not be discriminated within the TCD spectra^22^,^23^,^24^. The maximum number of bubbles in each case (i.e., either during normal breathing or during the VM) recorded in the MCA was taken as the estimate of shunt extent in the MCA. Estimates of shunt extent in the VA were obtained the same way. Tests were performed consecutively; each test required approximately 3 minutes, and there was an interval of at least 5 minutes from the last observed MB between tests.

Two ultrasound technologists were designated to assess the respective shunt extent of the MCA and VA in all subjects.

### Statistical analysis

Statistical analysis was performed with SPSS 17.0 software (SPSS Inc., Chicago, IL, USA). The chi-square test was used to compare positive rates of RLS detection. McNemar’s test was used to compare RLS detection by c-TCD between VA and MCA monitoring. Bowker’s test was used for comparisons of the shunt extents between VA and MCA. The time of the first MB occurrence between two different arteries was analyzed with the paired samples test. Statistical significance was set at p < 0.05.

## Additional Information

**How to cite this article**: Guo, Y.-Z. *et al.* Comparison of Vertebral Artery and Middle Cerebral Artery Monitoring for Right-to-left Shunt Detection by Contrast-enhanced Transcranial Doppler. *Sci. Rep.* **6**, 24932; doi: 10.1038/srep24932 (2016).

## Figures and Tables

**Table 1:** Demographic and clinical characteristics of the participants.

	Participants (n = 194)
Age (mean ± SD, years)	38 ± 12.6
Sex (Male/Female)	69/125
Symptoms or disease	
Headache	163
Dizziness	27
Ischemic stroke/ Transient ischemic attack	4

**Table 2:**
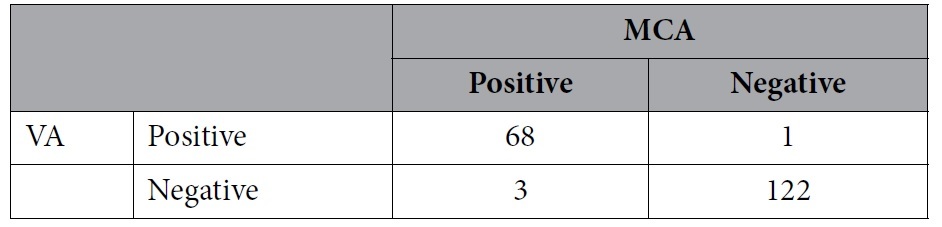
Comparison of right-to-left shunt detection by contrast-enhanced transcranial Doppler with vertebral artery (VA) and middle cerebral artery (MCA) monitoring.

Sensitivity 95.77%; specificity 99.19%; positive predictive value 98.55%; negative predictive value 97.60%.

p = 0.625.

**Table 3:**
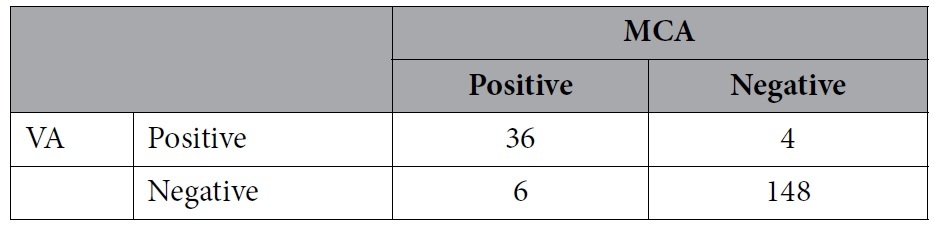
Comparison of constant right-to-left shunt detection by contrast-enhanced transcranial Doppler with vertebral artery (VA) and middle cerebral artery (MCA) monitoring.

Sensitivity 85.71%; specificity 97.37%; positive predictive value 90.00%; negative predictive value 96.10%.

p = 0.754.

**Table 4:**
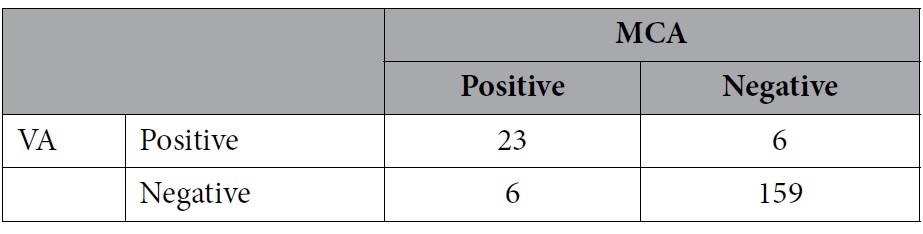
Comparison of provoked right-left-shunt detection by contrast-enhanced transcranial Doppler with vertebral artery (VA) and middle cerebral artery (MCA) monitoring.

Sensitivity 79.31%; specificity 96.36%; positive predictive value 79.31%; negative predictive value 96.36%.

p = 1.0.

**Table 5:**
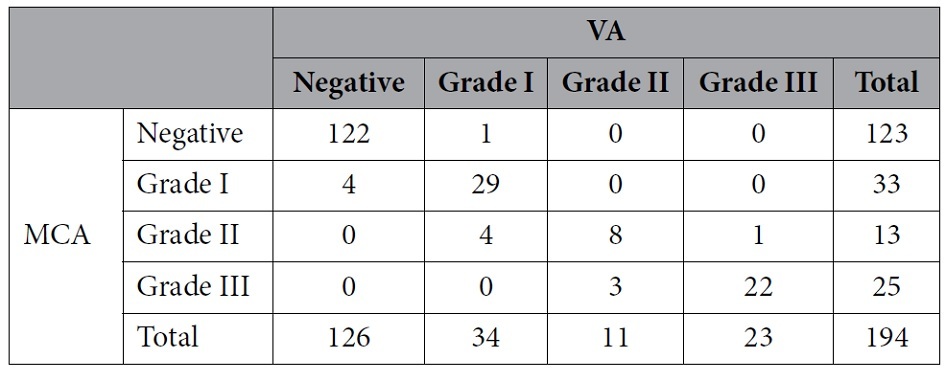
Different results of the extent of right-to-left shunt during vertebral artery (VA) and middle cerebral artery (MCA) monitoring.

p = 0.079.
